# Natural history of decompensated cirrhosis with serum hepatitis B DNA < 2000 IU/mL: a retrospective study

**DOI:** 10.1186/s12876-022-02541-1

**Published:** 2022-11-09

**Authors:** Xu Huang, Meimei Yan, Zerun Deng, Lei Yao, Dan Han, Lihua Sun

**Affiliations:** 1 Department of Epidemiology, The First Affiliated Hospital of Shandong First Medical University & Shandong Provincial Qianfoshan Hospital, No. 16766, Jingshi Road, Ji’nan City, Shandong Province 250000 People’s Republic of China; 2 Infectious Diseases Center, The First Affiliated Hospital of Xinjiang Medical University, No.137, Liyushan South Road830000, Urumqi City, Xinjiang Province People’s Republic of China

**Keywords:** Low HBV DNA levels, Hepatitis B virus, Decompensated cirrhosis, Retrospective study

## Abstract

**Background and aims:**

Patients with low HBV DNA levels (< 2000 IU/mL), HBV DNA negative, and HBsAg-negative hepatitis B virus(HBV)infection can still progress to decompensated cirrhosis; however, clinical research data in such patients, especially treatment-naïve patients, are currently insufficient. This study assessed the natural history of aforementioned patients.

**Methods:**

We retrospectively reviewed the data of 250 patients with HBV-associated decompensated cirrhosis(HBV DNA < 2000 IU/mL) who had not been treated with antiviral medication.

**Results:**

The mean age of the 250 patients was 53.90 ± 11.73 years and 183 patients (73.2%) were male. HBV DNA, HBsAg, and HBeAg positivity was detected in 77 (30.8%), 200 (80%), and 137 (54.8%) patients, respectively. HBsAg (odds ratio [OR], 3.303; 95% confidence interval [CI], 1.338–8.152; *P* = 0.010) and HBeAg (OR, 0.200; 95% CI, 0.107–0.376; *P* < 0.001) positivity were independent factors for low HBV DNA levels. The incidence of hepatocellular carcinoma (HCC) (*P* < 0.001) and portal vein thrombosis (*P* = 0.001) was higher in the low HBV DNA levels group. Multivariate analysis showed that HBV DNA positivity (OR, 3.548; 95% CI, 1.463–8.604; *P* = 0.005), HBeAg positivity (OR, 0.080; 95% CI, 0.022–0.289; *P* < 0.001), and glutamyltransferase (GGT) (OR, 1.003; 95% CI, 1.000–1.006; *P* = 0.040) were independent factors for HCC. Age was not related to the occurrence of cirrhosis complications.

**Conclusion:**

Patients with decompensated cirrhosis with HBV DNA < 2000 IU/mL still had severe liver damage and could develop severe cirrhosis complications. HCC risk was higher in low HBV DNA levels patients. HBsAg positivity and HBeAg negativity may be associated to the occurrence of low HBV DNA levels.

## Introduction

Hepatitis B virus (HBV) infection is a major global public health problem and the leading cause of liver cirrhosis and hepatocellular carcinoma (HCC) worldwide [1]. Liver cirrhosis is an important natural stage of HBV infection. Once infection progresses to cirrhosis, the incidence of HCC increases significantly, with an annual incidence up to 3%–6% [2–4]. Moreover, the 5-year survival rate of decompensated cirrhosis is only 14%–35% [5]. Now widely used, nucleos(t)ide analogue (NA), especially potent and low-resistance antiviral drugs such as entecavir, tenofovir, tenofovir alafenamide, can effectively inhibit viral replication, and long-term antiviral therapy can reverse liver cirrhosis [6, 7], reduce the incidence of HCC [8], reduce liver decompensation [9], and alter the natural history of decompensated cirrhosis [10].

However, some patients in clinical practice show low-level viremia (LLV) even after long-term treatment with potent NA. Although the definition of LLV has not been unified, it usually means that serum HBV DNA < 2000 IU/mL. Poor prognosis is strongly linked to LLV, especially in patients with cirrhosis. The REVEAL study showed an increased risk of cirrhosis and HCC with increased HBV DNA levels [11, 12]. LLV is also a possible cause of HCC in patients treated with NA [13, 14]. Our previous studies showed that patients with chronic hepatitis B (CHB) negative for HBV DNA and HBsAg can still progress to cirrhosis. The overall incidence of complications is not low in these patients (The overall incidence of severe complications in patients with serum HBV DNA < 2000 IU/mL, LLV, HBV DNA negative and HBsAg negative were 80%, 73.6%, 82.7% and 85.3%, respectively) and decompensation or HCC can occur [15].

Decompensated cirrhosis is the end stage of HBV infection, with significantly reduced survival time and quality of life causing a great burden on individuals and society. However, the few studies on decompensated cirrhosis of HBV DNA < 2000 IU/mL focused on the population receiving antiviral therapy. Therefore, an evaluation is required of the natural history of patients with HBV-associated decompensated cirrhosis with newly treated low HBV DNA levels, negative HBV DNA, and even HBsAg disappearance, to provide clinical evidence for evaluating such patients.

## Materials and methods

### Patients

This retrospective study included 250 patients with HBV-related decompensated cirrhosis(HBV DNA < 2000 IU/mL) and newly diagnosed admitted to the First Affiliated Hospital of Xinjiang Medical University between 2010 and 2021. The protocol for the study was approved by our institution's Ethics Committee. Owing to the retrospective design, the informed consent requirement was waived, and anonymized clinical data were analyzed. The inclusion criteria were: (1) Age ≥ 18 years; (2) HBV DNA concentration < 2000 IU/mL; (3) currently HBsAg positive or HBsAg negative, anti-HBc positive, and a clear history of chronic HBV infection (previous HBsAg positivity > 6 months), and other etiologies excluded; (4) cirrhosis diagnosed by clinical or imaging, and decompensation refers to serious complications such as esophagogastric variceal bleeding, ascites, and hepatic encephalopathy. The exclusion criteria were: (1) previous antiviral therapy; (2) a concomitant infection with other hepatitis viruses or human immunodeficiency virus (HIV); (3) history of liver transplantation; (4) the presence of other liver diseases such as alcoholic liver disease, autoimmune hepatitis, or drug-induced liver injury; (5) associated with other serious diseases such as other malignant tumors, connective tissue diseases, hematological diseases, other organ failure, or obstructive jaundice; and (6) incomplete data.

### Laboratory tests and imaging examinations

Data on virology, blood routine, biochemistry, coagulation function, and other related indicators of all patients were recorded. Serum alanine aminotransferase (ALT) and aspartate aminotransferase (AST) were set at 40 IU/L as the upper limit of normal. HBV DNA levels were quantified by quantitative fluorescent polymerase chain reaction (PCR) on an ABI 7300 real-time instrument (Applied Biosystems, USA) using a commercial kit for the detection of HBV nucleic acid (PCR-fluorescent probe method, Zhongshan Antu Bioengineering Co., Ltd, provided by Daan Gene Co., Ltd.) with a minimum detection limit of 100 IU/mL. The viral level of HBV DNA positivity was defined as 100–2000 IU/mL, whereas negativity was defined as < 100 IU/mL.HBsAg was detected using an AutoLumo A2000 Plus instrument and an HBV surface antigen quantitative detection kit (magnetic particle chemiluminescence method), all produced by Zhengzhou Antu Bioengineering Co., Ltd. Its linear range is 0.05–250 IU/mL.

Bleeding from esophagogastric varices was confirmed by gastroscopy and ascites, portal vein thrombosis, and HCC were diagnosed by computed tomography or magnetic resonance imaging.

### Statistical analysis

The data were analyzed using social science statistical program (SPSS 25.0). To assess the normality of continuous variables, Shapiro–Wilk tests were used. Normally distributed data are presented as means ± standard deviation and compared by independent-samples t-tests. Skewed data are presented as medians and interquartile range (P25, P75) by nonparametric U Mann–Whitney tests.

Categorical variables were represented by the number of cases n (%), and comparisons between groups were performed using chi-square tests. We compared clinical data for patients with and without low HBV DNA levels and HCC by univariate analysis and performed binary logistic regression on significant factors (*P* < 0.1) in univariate analysis.

## Results

### Patient characteristics

This study included 250 patients with an average age of 53.90 ± 11.73 years, of whom 183 patients were male (73.2%). In this study, 36 patients (14.4%) had hypertension, 48 patients (19.2%) had diabetes, 200 (80%) were HBsAg-positive, 137 (54.8%) were HBeAg-positive, and 77 (30.8%) were HBV DNA positive. The results of the laboratory tests showed increased levels of total bilirubin (TBIL) and aspartate aminotransferase (AST) and decreased levels of albumin (ALB) and platelets (PLT). Patients were stratified into grade A (38 patients, 15.2%) and grade B/C (212 patients, 84.8%) according to the Child-Turcotte-Pugh (CTP) score. The model of end-stage liver disease (MELD) score was 13.17 ± 6.13, while the median aspartate transferase and platelet ratio index (APRI) and liver fibrosis 4-factor index (FIB-4) were 1.98 (1.07, 3.27) and 6.69 (3.86, 9.91), respectively (Table 1).

**Table 1:** Clinical features of the study population

		All(*n* = 250)	HBV DNA-positive(*n* = 77)	HBV DNA-negative (*n* = 173)	*P*	HBsAg-positive(*n* = 200)	HBsAg-negative(*n* = 50)	*P*
Male sex		183,73.2	63,81.8	120,69.4	0.040	145,72.5	38,76.0	0.617
Age(y)		53.90 ± 11.73	54.13 ± 11.76	53.80 ± 11.75	0.837	53.01 ± 11.35	57.46 ± 11.65	0.016
Hypertension		36,14.4	12,15.6	24,13.9	0.722	25,12.5	11,22.0	0.087
Diabetes		48,19.2	14,18.2	34,19.7	0.785	40,20.0	8,16.0	0.521
CTP	A	38,15.2	8,10.4	30,17.3	0.158	25,12.5	13,26.0	0.017
	B/C	212, 84.8	69,89.6	143,82.7		175,87.5	37,74.0	
HBeAg-positive		137,54.8	23,29.9	114,65.9	< 0.001	107,53.5	30,60.0	0.409
HBsAg-positive		200, 80%	70,90.9%	130,75.1	0.004	-	-	-
HBV DNA-positive		-	-	-	-	70,35.0	7,14.0	0.004
MELD		13.17 ± 6.13	13.71 ± 5.89	12.92 ± 6.23	0.348	13.33 ± 6.32	12.52 ± 5.30	0.404
APRI		1.98(1.07, 3.27)	2.58(1.27, 3.57)	1.84(0.95,2.78)	0.005	2.10(1.17,3.27)	1.23(0.66, 2.37)	0.002
FIB-4		6.69(3.86, 9.91)	7.93(4.89, 10.85)	6.14(3.55,9.71)	0.044	7.42(4.45,9.79)	4.59(2.91,10.94)	0.050
TBIL(umol/L)		29.07(19.82,64.74)	30.90(20.94, 57.04)	29.00(16.66, 58.07)	0.301	28.85(17.78, 64.10)	29.71(21.58, 43.52)	0.942
ALB(g/L)		29.75(26.38,34.80)	29.58(26.65, 33.00)	30.19(25.66, 35.10)	0.495	29.58(26.24,34.18)	30.44(26.30,36.73)	0.084
SCr(umol/L)		62.00(51.22,77.00)	65.00(53.50,78.00)	61.00(49.00,74.83)	0.177	61.07(50.10,74.05)	64.38(52.98,81.02)	0.315
ALT(U/L)		33.43(21.65,66.12)	38.74(26.43, 75.73)	31.20(19.91, 56.32)	0.018	35.07(21.97,63.35)	27.95(17.62,45.29)	0.044
AST(U/L)		50.74(32.50,95.80)	62.40(35.49, 96.47)	46.00(30.15, 82.75)	0.071	53.33(35.03,92.50)	36.85(27.09,77.23)	0.051
GGT(U/L)		49.00(25.30,92.57)	55.00(32.73, 99.11)	46.00(23.39, 100.68)	0.214	49.15(26.65,91.09)	42.60(22.10,147.35)	0.765
ALP(U/L)		97.00(72.00,150.30)	109.10(76.60, 154.86)	93.00(70.52, 141.75)	0.108	100.36(76.20,147.65)	80.37(58.98, 156.78)	0.151
INR		1.35(1.15,1.55)	1.37(1.19,1.60)	1.33(1.15,1.58)	0.191	1.37(1.15,1.62)	1.28(1.13,1.45)	0.090
PLT(10^9/L)		68.00(46.00,113.50)	61(42.50,111.50)	72(46.00,123.00)	0.079	67.50(45.00,113.00)	80.50(49.50,139.25)	0.086

*Abbreviations*: *CTP* Child-Turcotte-Pugh, *MELD* Model for end-stage liver disease, *APRI* AST-to-platelet ratio index, *FIB 4* Fibrosis index based on four factors, *TBIL* Total bilirubin, *ALB* Albumin, *SCr* Serum creatinine, *ALT* Alanine aminotransferase, *AST* Aspartate aminotransferase, *GGT* Glutamyltransferase, *ALP* Alkaline phosphatase, *INR* International normalized ratio, *PLT* Platelet

Compared to the HBV DNA-negative group, the HBV DNA-positive group had more male patients (*P* = 0.040), more HBsAg positive (*P* = 0.004), less HBeAg positivity (*P* < 0.001), and FIB-4 (*P* = 0.044), APRI (*P* = 0.005), and ALT (*P* = 0.018) levels were greater in the HBV DNA-positive group. The HBsAg-positive group had a lower average age (*P* = 0.016), a higher proportion of CTP grades B/C (*P* = 0.017), and higher HBV DNA-positivity (*P* = 0.044) and APRI (*P* = 0.002) and ALT (*P* = 0.044) levels (Table 1).

### HBsAg and HBeAg positivity are independent factors for low HBV DNA levels

The independent factors for low HBV DNA levels were determined using multivariate analysis of the indicators (Table 1). HBsAg (odds ratio [OR], 3.303; 95% confidence interval [CI], 1.338–8.152; *P* = 0.010) and HBeAg (OR, 0.200; 95% CI, 0.107–0.376; *P* < 0.001) positivity were significant factors in predicting low HBV DNA levels in patients with HBV-related cirrhosis (Table 2).

**Table 2:** Multivariate analysis for low HBV DNA levels

	Multivariable model		
	OR	95% CI	*P*
Male sex	1.781	0.859–3.696	0.121
HBsAg-positive	3.303	1.338–8.152	**0.010**
HBeAg-positive	0.200	0.107–0.376	** < 0.001**
APRI	1.009	0.892–1.142	0.885
FIB-4	1.005	0.958–1.054	0.830
ALT	1.001	0.997–1.006	0.522
AST	1.001	0.995–1.006	0.841
PLT	0.995	0.990–1.000	0.072

*Abbreviations*: *APRI* AST-to-platelet ratio index, *FIB 4* Fibrosis index based on four factors, *ALT* Alanine aminotransferase, *AST* Aspartate aminotransferase, *PLT* Platelet

### Relationship between HBV DNA, HBsAg, and complications of liver cirrhosis

In this study, 250 patients developed severe complications of liver cirrhosis, including ascites (217, 86.8%), esophagogastric variceal bleeding (65, 26.0%), HCC (35, 14.0%), portal vein thrombosis (29, 11.6%), hepatic encephalopathy (13, 5.2%), and hepatorenal syndrome (4, 1.6%). In patients with low HBV DNA levels, even those negative for HBV DNA and HBsAg, the incidence of cirrhosis-related complications remained high; therefore, regardless of the levels of HBV DNA and HBsAg, it is also important to reduce portal pressure while actively administering antiviral therapy to reduce the occurrence of complications (Table 3).

**Table 3:** Incidence of cirrhosis-related complications

	**All(*n* = 250)**	**HBV DNA-positive** **(*n* = 77)**	**HBV DNA-negative** **(*n* = 173)**	***P***	**HBsAg-positive(*n* = 200)**	**HBsAg-negative(*n* = 50)**	***P***
Ascites	217,86.8	69,89.6	148,85.5	0.381	180,90.0	37,74.0	**0.003**
EVB	65,26.0	18,23.4	47,27.2	0.528	47,23.5	18,36.0	0.071
HCC	35,14.0	23,29.8	12,6.9	** < 0.001**	31,15.5	4,8.0	0.172
PVT	29,11.6	17,22.1	12,6.9	**0.001**	21,10.5	8,16.0	0.277
HE	13,5.2	6,7.8	7,4.0	0.356	12,6.0	1,2.0	0.433
HRS	4,1.6	1,1.3	3,1.7	1.000	4,2.0	0,0	0.705

*Abbreviations*: *EVB* Esophagogastric variceal bleeding, *HE* Hepatic encephalopathy, *HRS* Hepatorenal syndrome, *HCC* Hepatocellular carcinoma, *PVT* Portal vein thrombosis

We compared the incidence of complications between patients with HBV DNA and HBsAg positivity and negativity.The incidence of HCC (*P* < 0.001) and portal vein thrombosis (*P* = 0.001) was higher in the HBV DNA-positive group, while the incidence of ascites was greater in the HBsAg-positive group (*P* = 0.003) (Table 3).

### Low HBV DNA levels is an independent risk factor for HCC

In this study, 35 patients (14.0%) had HCC. Table 3 shows that the incidence of HCC was greater in the HBV DNA-positive group. Univariate analysis showed that male sex (*P* = 0.009), HBV DNA positivity (*P* < 0.001), HBeAg positivity (*P* < 0.001), GGT (*P* = 0.001),and ALT (*P* = 0.001) were related with the occurrence of HCC. Binary logistic regression analysis of factors with *P* < 0.1 in univariate analysis showed that HBV DNA positivity (OR, 3.548; 95% CI, 1.463–8.604; *P* = 0.005), HBeAg positivity (OR, 0.080; 95% CI, 0.022–0.289; *P* < 0.001), and GGT (OR, 1.003; 95% CI, 1.000–1.006; *P* = 0.040) were independent factors for HCC (Table 4).

**Table 4:** Univariate and multivariate analysis of the occurrence of HCC

	Univariate *P* Value	Multivariable model		
		OR	95% CI	*P*
Age	0.364			
Male sex	0.009	2.619	0.698–9.821	0.153
HBV DNA-positive	< 0.001	3.548	1.463–8.604	**0.005**
HBsAg-positive	0.172			
HBeAg-positive	< 0.001	0.080	0.022–0.289	** < 0.001**
Diabetes	0.085	0.292	0.079–1.085	0.066
ALT	0.001	1.000	0.997–1.004	0.794
GGT	0.001	1.003	1.000–1.006	**0.040**

*Abbreviations*: *ALT* Alanine aminotransferase, *GGT* Glutamyltransferase

### Relationship between age and complications of liver cirrhosis

We divided the 250 patients according to their ages into three groups: < 40 years (25 patients), 40–50 years (75 patients), and > 50 years (150 patients). All age groups had the same incidence of complications (*P* > 0.05) (Table 5).

**Table 5:** Complications in different age groups

	**All(*n* = 250)**	** < 40 years(*n* = 25)**	**40–50 years(*n* = 75)**	** > 50 years (*n* = 150)**	***P***
EVB	65,26.0	10,40.0	18,24.0	37,24.7	0.242
Ascites	217,86.8	23,92.0	65,86.7	129,86.0	0.685
HE	13,5.2	0,0	3,4.0	10,6.7	0.173
HRS	4,1.6	0,0	0,0	4,2.7	0.127
HCC	35,14.0	2,8.0	10,13.3	23,15.3	0.575
PVT	29,11.6	2,8.0	7,9.3	20,13.3	0.557

*Abbreviations*: *EVB* Esophagogastric variceal bleeding, *HE* Hepatic encephalopathy, *HRS* Hepatorenal syndrome, *HCC* Hepatocellular carcinoma, *PVT* Portal vein thrombosis

## Discussion

The internationally recommended anti-HBV drugs include NA and polyethylene glycol interferon (PEG-IFN). NA have high potency, low drug resistance, and few adverse reactions and have become the first-line treatment drugs; however, they cannot completely remove covalently closed circular DNA (cccDNA) in hepatocytes; thus, it is difficult to achieve a complete cure. LLV occurs in 20–40% of patients even with first-line drugs [13, 16, 17]. The negative conversion rate of HBsAg is very low when interferon is used alone or in combination with NA [18]. As LLV is strongly associated with adverse outcomes, complete virological response of CHB is very important. In fact, after standardized antiviral therapy, some patients can still progress to decompensated liver cirrhosis and develop liver cirrhosis-related complications and HCC, regardless of low HBV DNA levels, HBV DNA negativity, or HBsAg disappearance. Few studies have reported on HBV-associated decompensated cirrhosis in patients with LLV, especially those who have not received antiviral treatment. This study retrospectively analyzed the natural history of decompensated cirrhosis patients with HBV DNA < 2000 IU/mL who had not been treated with antiviral medication.

We found that, among decompensated cirrhosis patients with HBV DNA < 2000 IU/mL, most were middle-aged and elderly men. Most patients were HBeAg-positive and HBV DNA-negative, and 62 patients (20.6%) had HBsAg negative conversion. Previous studies have shown that high HBV DNA levels, persistent HBeAg positivity, persistent ALT elevation, advanced age, and male sex are risk factors for cirrhosis [19–21], consistent with the results of this study. However, we found that patients with low HBV DNA levels, HBV DNA negativity, and HBsAg negativity can advance to decompensated cirrhosis and even develop HCC. In addition, the liver damage in these patients is also severe, TBIL and AST were increased, and the ALB and PLT levels were decreased. Moreover, the MELD, APRI, and FIB-4 scores were increased. Therefore, after these patients progress to decompensated cirrhosis, even if the virus replication level is very low and HBsAg disappears, the liver function is still damaged. This damage does not manifest as liver inflammatory damage characterized by significant elevation of ALT or AST, nor as liver failure characterized by the rapid progression of high jaundice and low blood coagulation, but rather as a slow and continuous progression of liver fibrosis and progressive elevation of portal hypertension, which ultimately develop into recurrent complications and cancer due to portal hypertension. Therefore, in addition to more effective antiviral treatment, these patients also require evaluation and intervention for portal hypertension.

Multivariate analysis to explore the factors affecting the occurrence of low HBV DNA levels showed that HBsAg positivity and HBeAg positivity were independent factors for low HBV DNA levels. A Korean study reported a significantly higher cumulative complete virological response rate (CVR) in the HBeAg-negative group in patients with CHB receiving tenofovir anti-viral treatment(*P* = 0.001). Moreover, HBsAg quantification were significant predictors of CVR(*P* < 0.001) [22]. Another retrospective study reported that HBeAg status was related to LLV [13]. Thus, HBsAg and HBeAg statuses may be related to LLV and have a certain value for predicting LLV during antiviral treatment.

We observed that patients with low HBV DNA levels had higher APRI and FIB-4 scores and a higher incidence of HCC and portal vein thrombosis(PVT). The degree of liver fibrosis and the incidence of complications of liver cirrhosis in HBV DNA-negative patients were lower than those in HBV DNA-positive patients, with lower virus levels related to a better status. A longitudinal study of 163 treatment-naïve patients with CHB with significant fibrosis at baseline (Ishak ≥ 3) showed that patients with LLV had a 4.84-fold higher risk of liver fibrosis progression at 78 weeks of treatment compared to HBV DNA-negative patients(95% CI, 1.30–17.98; *P* = 0.019), indicating that LLV was an independent predictor of liver fibrosis progression [23]. Another retrospective study from Turkey also observed progression to liver fibrosis in approximately 1/3 of young patients with LLV, suggesting that even young patients with LLV may have an affected prognosis [24]. A multicenter prospective study showed that, among patients with HBV-related decompensated cirrhosis, the 10-year survival rate of patients with a maintained virological response (MVR) was 71.6%, whereas that of the LLV group was only 57.2% (*P* < 0.003). Thus, LLV contributed to reduced long-term survival in patients with decompensated cirrhosis [10]. A recent study of patients with cirrhosis treated with NA reported 5-year and 10-year cumulative incidence rates of end-stage liver disease in the LLV group of 13.75% and 36.89%, respectively, while that in the MVR group was 2.66% and 21.29%, suggesting that NA treatment of patients with cirrhosis and LLV may result in worse long-term outcomes than MVR [25]. In this study, the incidence of HCC and PVT was higher in the low HBV DNA levels group, while the remaining complications did not differ significantly, which may be related to the fact that none of the patients included in this study had received antiviral treatment. In such patients, cccDNA persists and low HBV DNA levels may appear intermittently, which promotes the progression of liver inflammation and fibrosis. After progressing to decompensated cirrhosis, even without detectable HBV DNA, severe liver fibrosis and severe liver damage have developed and complications related to liver cirrhosis can still occur. Therefore, low HBV DNA levels patients with cirrhosis still require active antiviral therapy to suppress viral replication as much as possible to reduce the incidence of related complications and improve prognosis.

In addition, the mean age of the 50 HBsAg-negative patients in this study was older than that of HBsAg-positive patients; however, complications other than ascites did not differ significantly from those of HBsAg-positive patients. Various complications may still occur after HBsAg serological clearance and the incidence does not differ significantly from that of HBsAg-positive patients, even HCC, suggesting that HBsAg seroclearance can reduce HCC risk among patients with liver cirrhosis [26]. Another study reported that 2.34% (7/298) of patients with HBsAg serological clearance developed HCC during the 9-year median follow-up period [27]. The incidence of HCC in HBsAg-negative patients in this study was 8% (4/50), a significantly higher rate than previously reported. This difference may have been related to decompensated cirrhosis in the patients included in this study. Therefore, for decompensated cirrhosis, even if HBsAg disappears, the incidence of cirrhosis-related complications remains high, and regular follow-up examinations are required to prevent related complications.Moreover, among the 50 patients with HBsAg-negative cirrhosis in this study, seven (14%) were low HBV DNA levels and had occult hepatitis B infection (OBI), one of whom developed HCC. Therefore, for patients with liver cirrhosis negative for HBsAg, whether they have anti-HBc or anti-HBs, HBV DNA should be observed to rule out OBI and allow timely treatment to prevent HCC.

Age was previously considered a high-risk factor for liver cirrhosis. In our study, 150 patients (60%) were > 50 years of age; however, the incidence of complications did not differ significantly from that in other age groups. During chronic and long-term HBV infection repeated immune clearance responses lead to frequent hepatic inflammatory activity; thus, even if HBV DNA and HBsAg are negative, severe liver fibrosis is likely present due to late occurrence and liver-related adverse events can still occur. Therefore, once low HBV DNA levels patients in the natural state progresses to decompensated cirrhosis, age is no longer a prognostic factor. Patients with HBV-associated decompensated liver cirrhosis, regardless of age and virological indicators, show a high incidence of liver cirrhosis-associated complications; thus, complications should be monitored in clinical practice.

Liver cirrhosis is a significant risk factor for HCC and higher HBV DNA levels are associated with increased HCC risk [28]. A cohort study in Hong Kong reported a higher risk of developing HCC within 2 years in patients with CHB with previous NA treatment and serum HBV DNA concentrations of 10–20 IU/mL compared to patients with CHB with HBV DNA concentrations < 10 IU/mL (hazard ratio [HR] 2.79, 95% CI 1.424–5.468), suggesting that even a very low virus level after antiviral therapy still increases the risk of HCC [29]. A Korean study of patients with compensated HBV-related cirrhosis without antiviral treatment reported a higher risk of HCC in the LLV group compared to that in the group with continuous complete virological response(CVR) [14]. Moreover, the HCC risk increased with LLV after treatment in patients with cirrhosis [13, 30]. The results of another Korean retrospective cohort study showed that approximately 27.8% of patients with HCC with LLV developed HBV relapse during follow-up, and that the 5-year survival rate of HBV relapsed patients was significantly lower than that of patients who achieved and sustained CVR [31]. However, a prospective study reported in patients with HBV-associated decompensated cirrhosis after receiving antiviral treatment 5-year and 10-year cumulative incidence rates of HCC in the LLV group of 39.5% and 43.9% respectively, compared to 22.9% and 41.0% in the continuous virological response group. The incidence rates of HCC did not differ significantly between the two groups (*P* = 0.195) [32]. Therefore, the relationship between LLV and HCC in patients with decompensated cirrhosis remains unclear. We observed a significantly higher incidence of HCC in the low HBV DNA levels group than that in the HBV DNA-negative group and identified low HBV DNA levels as an independent risk factor for HCC in multivariate analysis. The current treatment guidelines for patients with decompensated cirrhosis recommend antiviral treatment regardless of viral level [28, 33, 34]. However, we cannot ignore the virological response of such patients because they may benefit from a sustained virological response.

We also observed that HBeAg-negative patients had a greater risk of HCC and that HBeAg-negativity was an independent risk factor for HCC. Previous studies showed a reduced risk of HCC in patients with CHB after spontaneous HBeAg seroconversion [35]. Another multinational cohort study showed that HBeAg positivity was not associated with the risk of HCC in patients with cirrhosis [36]. Moreover, age > 40 years at the time of HBeAg seroconversion was associated with a higher risk of HCC [19, 37]. In our study, 90% of patients (225/250) were > 40 years. Thus, the higher risk of HCC in HBeAg-negative patients may be associated with their age at the time of HBeAg seroconversion. The occurrence of HCC should be considered in patients with CHB who are > 40 years of age, regardless of the HBV DNA, HBeAg, and HBsAg status.

This study has some limitations. First, as a single-center retrospective study, the findings require confirmation in multi-center prospective studies with larger sample sizes and the degree of benefit of antiviral therapy must be evaluated in this population. And as high-sensitivity PCR was not previously performed, 100 IU/mL was selected as the lower limit of detection. Therefore, while some HBV DNA-negative patients may have had low HBV DNA levels, their presence did not affect the analysis and final conclusions.Second, this study was conducted in a tertiary hospital. As the included patients may be more critically ill, selection bias was possible. Moreover, some complications such as mild hepatic encephalopathy may be missed, which reduces the incidence of complications, due to the retrospective design, Additionally, this study did not consider the influence of other medical factors, particularly metabolic and lifestyle factors. Finally, none of the patients in this study had received antiviral therapy. Further research is needed to identify any potential differences in these patients after antiviral therapy.

In conclusion, the results of this study showed that liver damage of decompensated cirrhosis in patients with HBV DNA < 2000 IU/mL remains a serious concern and that related complications can still occur. Moreover, patients with low HBV DNA levels have an increased risk of HCC. HBsAg-positivity and HBeAg-negativity may be related to the occurrence of low HBV DNA levels. The prognosis of such patients is poor, and antiviral therapy is provided mainly to suppress the virus level to the maximum extent. The disappearance of HBsAg may not be related to long-term prognosis. Therefore, attention should be paid to the control of portal hypertension in this population.

## Data Availability

The datasets used and/or analyzed during the current study will be available from the corresponding author on reasonable request.

## Abbreviations

HBV: Hepatitis B virus
HCC: Hepatocellular carcinoma
NA: Nucleos(t)ide analogue
LLV: Low-level viremia
CHB: Chronic hepatitis B
CTP: Child-Turcotte-Pugh
MELD: Model for end-stage liver disease
APRI: AST-to-platelet ratio index
FIB-4: Fibrosis index based on four factors
TBIL: Total bilirubin
ALB: Albumin
SCr: Serum creatinine
ALT: Alanine aminotransferase
AST: Aspartate aminotransferase
GGT: Glutamyltransferase
ALP: Alkaline phosphatase
INR: International normalized ratio
PLT: Platelet
EVB: Esophagogastric variceal bleeding
HE: Hepatic encephalopathy
HRS: Hepatorenal syndrome
PVT: Portal vein thrombosis
